# Automated image-based assay for evaluation of HIV neutralization and cell-to-cell fusion inhibition

**DOI:** 10.1186/1471-2334-14-472

**Published:** 2014-08-30

**Authors:** Enas Sheik-Khalil, Mark-Anthony Bray, Gülsen Özkaya Şahin, Gabriella Scarlatti, Marianne Jansson, Anne E Carpenter, Eva Maria Fenyö

**Affiliations:** Department of Laboratory Medicine, Lund University, Lund, Sweden; Broad Institute of Harvard and MIT, Imaging Platform, Cambridge, MA USA; Viral Evolution and Transmission Unit, San Raffaele Scientific Institute, Milan, Italy; Department of Clinical Microbiology and Immunology, Lund University, Lund, Sweden

**Keywords:** Automated plaque reduction assay (APR assay), Fluorescence, HIV, Neutralization, Fusion inhibition

## Abstract

**Background:**

Standardized techniques to detect HIV-neutralizing antibody responses are of great importance in the search for an HIV vaccine.

**Methods:**

Here, we present a high-throughput, high-content automated plaque reduction (APR) assay based on automated microscopy and image analysis that allows evaluation of neutralization and inhibition of cell-cell fusion within the same assay. Neutralization of virus particles is measured as a reduction in the number of fluorescent plaques, and inhibition of cell-cell fusion as a reduction in plaque area.

**Results:**

We found neutralization strength to be a significant factor in the ability of virus to form syncytia. Further, we introduce the inhibitory concentration of plaque area reduction (ICpar) as an additional measure of antiviral activity, i.e. fusion inhibition.

**Conclusions:**

We present an automated image based high-throughput, high-content HIV plaque reduction assay. This allows, for the first time, simultaneous evaluation of neutralization and inhibition of cell-cell fusion within the same assay, by quantifying the reduction in number of plaques and mean plaque area, respectively. Inhibition of cell-to-cell fusion requires higher quantities of inhibitory reagent than inhibition of virus neutralization.

**Electronic supplementary material:**

The online version of this article (doi:10.1186/1471-2334-14-472) contains supplementary material, which is available to authorized users.

## Background

The assessment of virus specific antibodies plays a central role in human immunodeficiency virus type 1 (HIV-1) vaccine development [1]. Thus, evaluation of HIV/AIDS preventive vaccines requires the development of methods to assess different properties, including neutralizing capacity, of protective antibody responses. However, no single assay has so far been reported to detect such protective antibodies. Instead, a wide range of HIV-1 neutralization assays and variants thereof have been developed and described in the literature [2, 3]. These assays are based on different technologies but they all rely on the principle to measure reduction of virus infectivity in susceptible cells in the presence of inhibitory reagents. Likewise, the plaque reduction assay (PR) is based on virus infectivity and uses human cell lines (U87.CD4 or GHOST(3)) engineered to express HIV receptors [4, 5].

In parallel, major advances in high-throughput fluorescence microscopy and automated, high-content image analysis tools have paved the way for systematic and quantitative study of biological systems [6–10]. Fluorescence-based imaging assays have been applied to large-scale analysis to solve biological problems.

Here, we describe an automated PR assay, including sample preparation, automated image acquisition, and a computational image analysis pipeline using open-source software in order to convert the PR assay for HIV neutralization into a high-throughput, high-content assay. In addition to quantifying neutralization by reduction of plaque number, our high-content assay measures plaque area, permitting studies of cell-cell fusion inhibition. We compare this assay with manual readout and with other neutralization assays using a reference panel of inhibitory reagents, i.e. plasma and monoclonal antibodies. We demonstrate that by the use of image analysis the APR assay is converted to a high-throughput and high-content assay, where plaque area is a measure of cell-cell fusion.

## Methods

### Plaque Reduction (PR) assay

Infection assays were done by using HIV-1 isolates (of different subtypes and coreceptor use) and polyclonal inhibitory reagents (eight HIV-positive and one HIV-negative plasma), obtained from the Centre for AIDS Reagents (CFAR) NIBSC, UK, as previously described [3]. We tested virus neutralization in GHOST(3)-CCR5 and -CXCR4 cell lines stably transfected with CD4, chemokine receptors and the Tat-inducible green fluorescence protein (GFP) [4, 11]. Three days following exposure of cells to the plasma-virus mixtures, plaque-forming units (PFU) under the fluorescent microscope are counted to calculate neutralization as percentage of plaque reduction (PR) in the sample containing inhibitory reagent: [1 - (PFU with inhibitory reagent/PFU without inhibitory reagent)] × 100 [5]. For the manual readout the plaques were counted by eye in separate experiments.

### Automated Plaque Reduction assay (APR assay)

**Figure 1:**
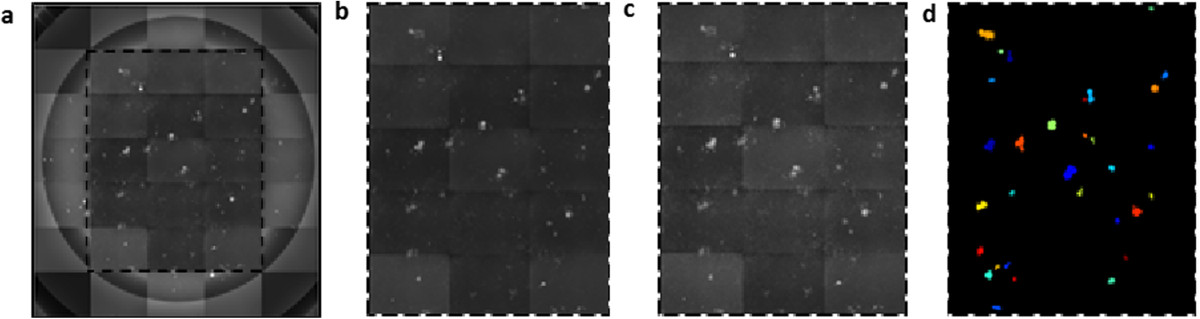
**The APR assay pipeline tiles the inner 15 images from each well (a) into a mosaic (b).** The pipeline includes algorithms for illumination correction **(c)** and plaque identification **(d)**. The colors labeling the plaques are used for illustrative purposes and chosen arbitrarily by the program.

**Figure 2:**
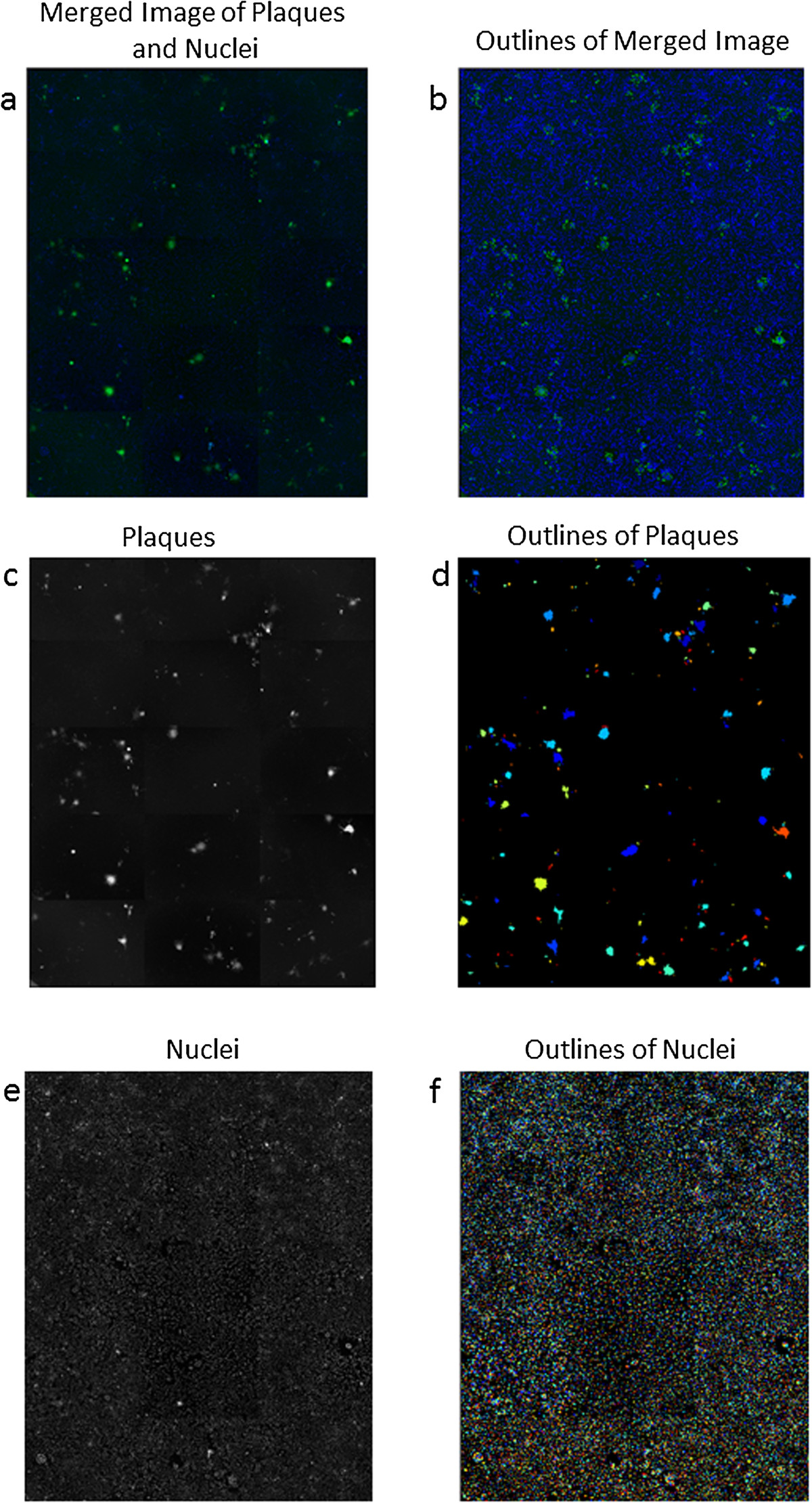
**Mosaic image of a well (a-c-e) is shown along with outlines (b-d-f) as defined by the borders of plaques and nuclei set by the APR threshold program.** All images are taken in the same microscopic field. Plaques (green) and nuclei (blue) are shown as a merged image **(a)** or plaques alone **(c)** or all nuclei **(e)**. The green color labeling of the outlines is used for illustrative purposes only.

**Figure 3:**
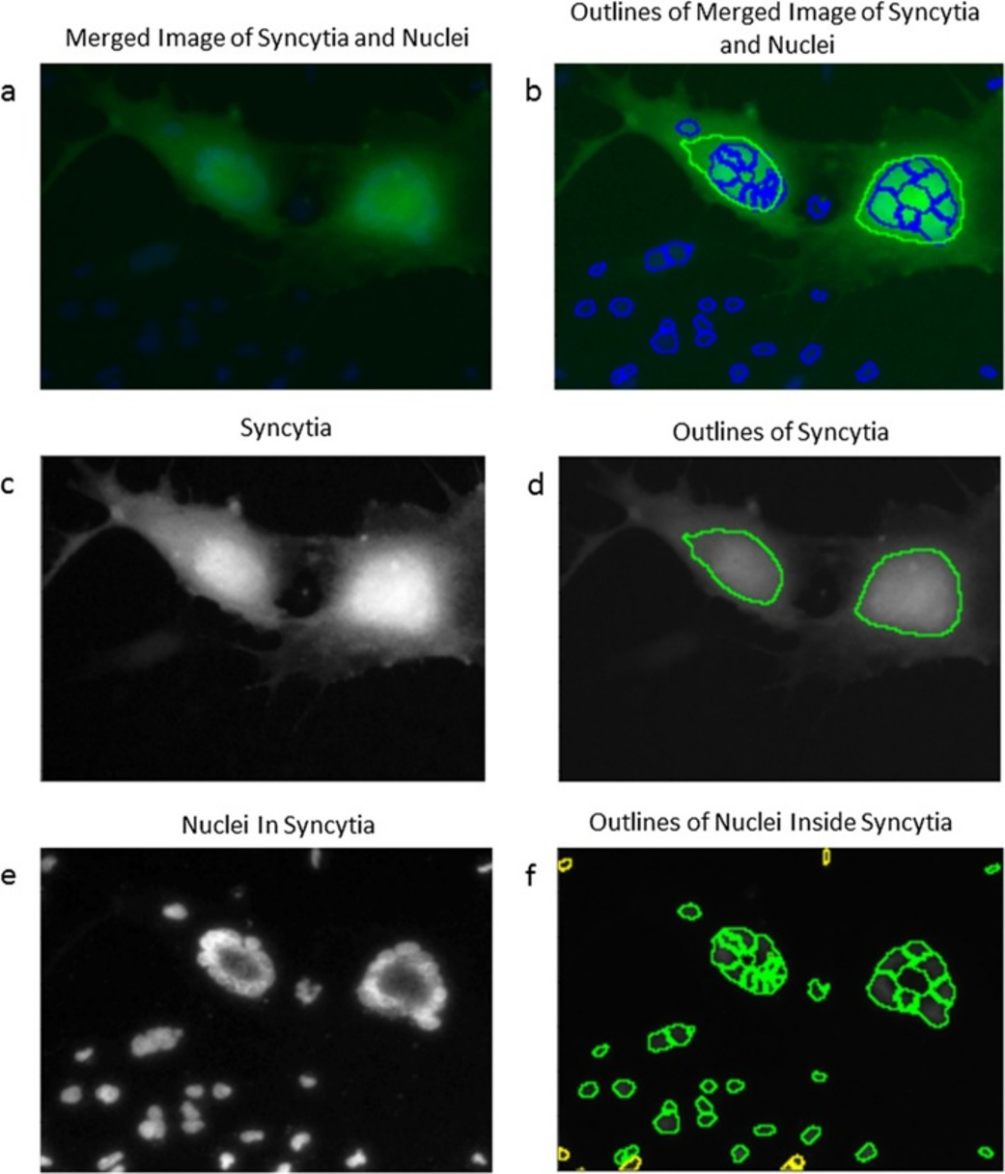
**A magnified picture of syncytia (green) and nuclei (blue) is shown as a merged image (a) along with the outlines (b).** Image of syncytia **(c)** along with the outlines **(d)** as well as nuclei inside the syncytia **(e)** with the outlines **(f)** clearly show how nuclei cluster inside syncytia. The green color labeling the outlines is used for illustrative purposes only.

**Figure 4:**
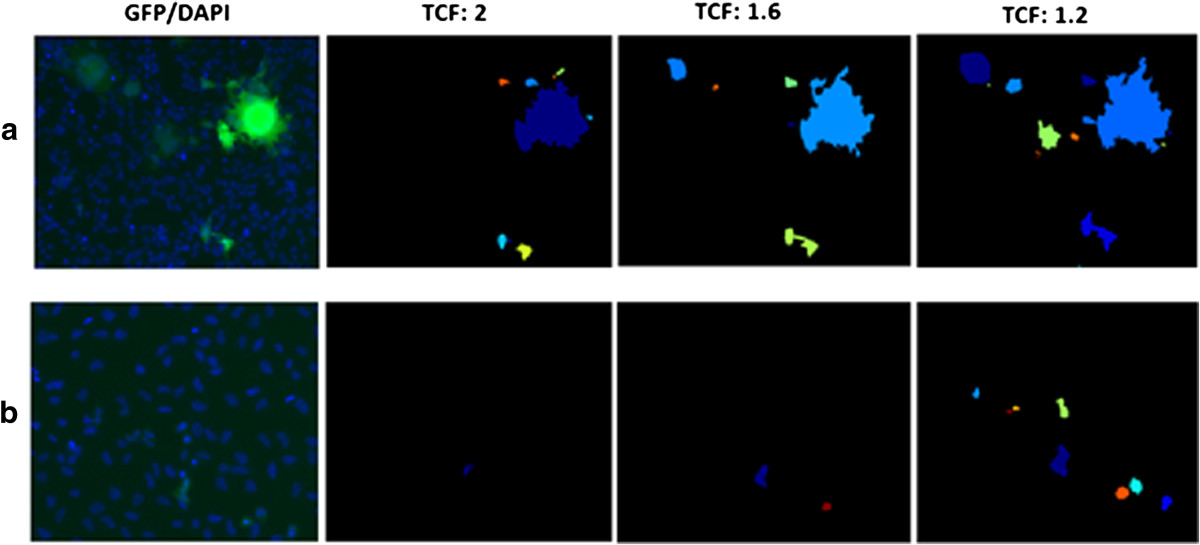
**Effect of the threshold correction factor (TCF) on plaque detection in GHOST(3)- CCR5/CXCR4 cells for (a) HIV-1-infected and (b) uninfected cases.** The TCF is located in the IdentifyPrimaryObjects module in the APR image analysis pipeline and affects the stringency or leniency of foreground/background thresholding. Column 1: Fluorescence microscopy images with HIV-1-infected cells expressing GFP (green) and nuclei stained with DAPI (blue). Columns 2 – 4: GFP-expressing syncytia detected for various values of the TCF, as noted in the column title. The colors labeling the syncytia are used for illustrative purposes and chosen arbitrarily.

To develop the assay in this study, we adapted the prior protocol for the manual PR assay [4] in 96-well plates and modified the readout by treating cells with Hoechst 33342 for 20 minutes, washing with PBS, and sealing plates with aluminum foil prior to automated image acquisition (Supp. Note 1). For the image acquisition, we used the AxioObserver Z1 with a Zeiss Neofluar Objective 10X/0.3 with the exposure time 200 ms. The inner 5 × 3 images of each well (approx. 4.108 mm × 5.117 mm) were imaged, to be tiled later into a single mosaic image. Assay quantification was performed by developing an APR workflow using the freely available image analysis software CellProfiler (http://www.cellprofiler.org) [12, 13]. The workflow consists of two pipelines comprised of image processing modules optimized to identify GHOST(3)-CXCR4 and GHOST(3)-CCR5 cells where HIV infection has activated the GFP marker. The first pipeline tiles the inner 5 × 3 image sites of each well into a single mosaic (approx. 4.108 mm × 5.117 mm) so as not to overestimate by counting plaques that lie on the image edge twice. A secondary purpose for this pipeline is to reduce background fluorescence and illumination heterogeneities present in the images prior to tiling, plaque detection and measurement (Figure 1). The second pipeline is used for plaque identification and measurement of parameters such as total cell and plaque count, plaque area, fluorescence intensity and morphology (Figure 2). In order to assess cell viability, nuclei are also identified and counted based on the DAPI stained images. Syncytia are formed due to merging of multiple cells (Figure 3). Several parameters in this pipeline can be fine-tuned for plaque identification; the effect of altering one such parameter is demonstrated in Figure 4. Detailed instructions on the use of the PR workflow is described in the Additional file 1 and provided online (http://www.cellprofiler.org/published_pipelines.shtml); other optimization considerations are provided in the module notes that may be viewed when the pipelines are loaded in CellProfiler.

### Details of plaque determination

As is the case for many biological applications, the most challenging bottleneck in configuring an image analysis pipeline is the segmentation, defined as the identification and partitioning of the individual plaques in the image. The IdentifyPrimaryObjects module in the APR image analysis pipeline was adjusted to read foreground/background to match manual reading. The results of the automatic readout and what would be the corresponding manual readout within the same experiment are thus closely similar, if not identical.

Here we describe several key settings contained in the IdentifyPrimaryObjects module in the plaque identification pipeline; these are also described in the module notes within the CellProfiler user interface:

- *Choice of automatic thresholding method:* In this context, thresholding refers to the use of intensity values to distinguish the image foreground (i.e., the GFP-expressing plaques) from the background. A number of automatic thresholding methods are provided for use in the module; the thresholding method chosen must not only reliably identify bright distinct plaques but also the smaller, dim single cells in order to be sufficiently sensitive. For the PR assay, the “Robust Background” thresholding method was used, which assumes a Gaussian intensity distribution after trimming the brightest and dimmest 5% of pixel intensities; the threshold is then calculated as the mean of this distribution plus 2 standard deviations.
- *Correction of the threshold value:* If the automatically determined threshold is consistently too high or too low in all images, it can be further refined by adjusting a module setting which multiplies the threshold by a constant value (“threshold correction factor”). Our automatic readout was performed after fine-tuning the threshold correction factor (TCF) on plaque detection in GHOST(3)- CCR5/CXCR4 cells for HIV-1-infected and uninfected cases (as explained in the legend for Figure 4). This selection was made manually by careful review of many plates and pictures. and verified through comparison of uninfected and HIV-1 infected GHOST(3) cultures. Care is required in correction factor selection since an excessively high value will identify only the brightest plaques (i.e., yield false negatives) and underestimate the plaque area in GFP-positive images, whereas a value that is too low will detect false positives in GFP-negative images (Figure 4). A threshold correction factor of 1.6 was found to be optimal for this assay.
- *Selection of the lower threshold limit:* In instances where the image is GFP-negative (i.e., no plaques), the automatic threshold value may be too low and therefore detects false positives. A lower threshold bound can be set as a precautionary measure by empirically estimating the GFP-negative signal across several images. For the PR assay, the lower threshold bound was set to 0.013.
- O*bject exclusion based on plaque size:* The upper and lower bounds for the typical diameter of a GFP-identified plaque can be adjusted to exclude spurious foreground regions, thereby precluding false positives. This size range was set to 4–150 pixels after calculating the mean diameter of a number of individual GHOST(3) cells in brightfield mode (data not shown).

### PBMC- and pseudovirus- based neutralization assays

The detailed methodologies of the peripheral blood mononuclear cell (PBMC)- and Env (gp160) pseudotyped virus (PSV)-based neutralization assays are available on the EUROPRISE website (http://www.europrise.org/neutnet_sops.html). In brief, seven laboratories performed the PBMC-based assay, where virus isolates were used with PBMC (isolated from buffy coats of HIV-negative blood donors) as target cells. The PSV-based assay was performed by six different laboratories as a single cycle assay and by one laboratory as a multiple cycle infection assay with engineered cell lines as target cells.

### Statistics

The non-parametric Spearman rank statistical analysis was used to calculate correlations between findings of the automated and manual plaque reduction assays and neutralization to change in plaque area. For comparison of the three different neutralization assays in relation to virus neutralization sensitivity and plasma neutralization capacity, as well as the mean plaque area of different uninhibited HIV-1 isolates the non-parametric Kruskal Wallis test, with Dunn’s post test, was used. Normalized mean plaque area for virus-plasma combinations, at IC50, IC75 and IC90, was evaluated according to Friedman’s test and Dunn’s Post Test.

## Results

### Comparison of manual and APR readout

**Figure 5:**
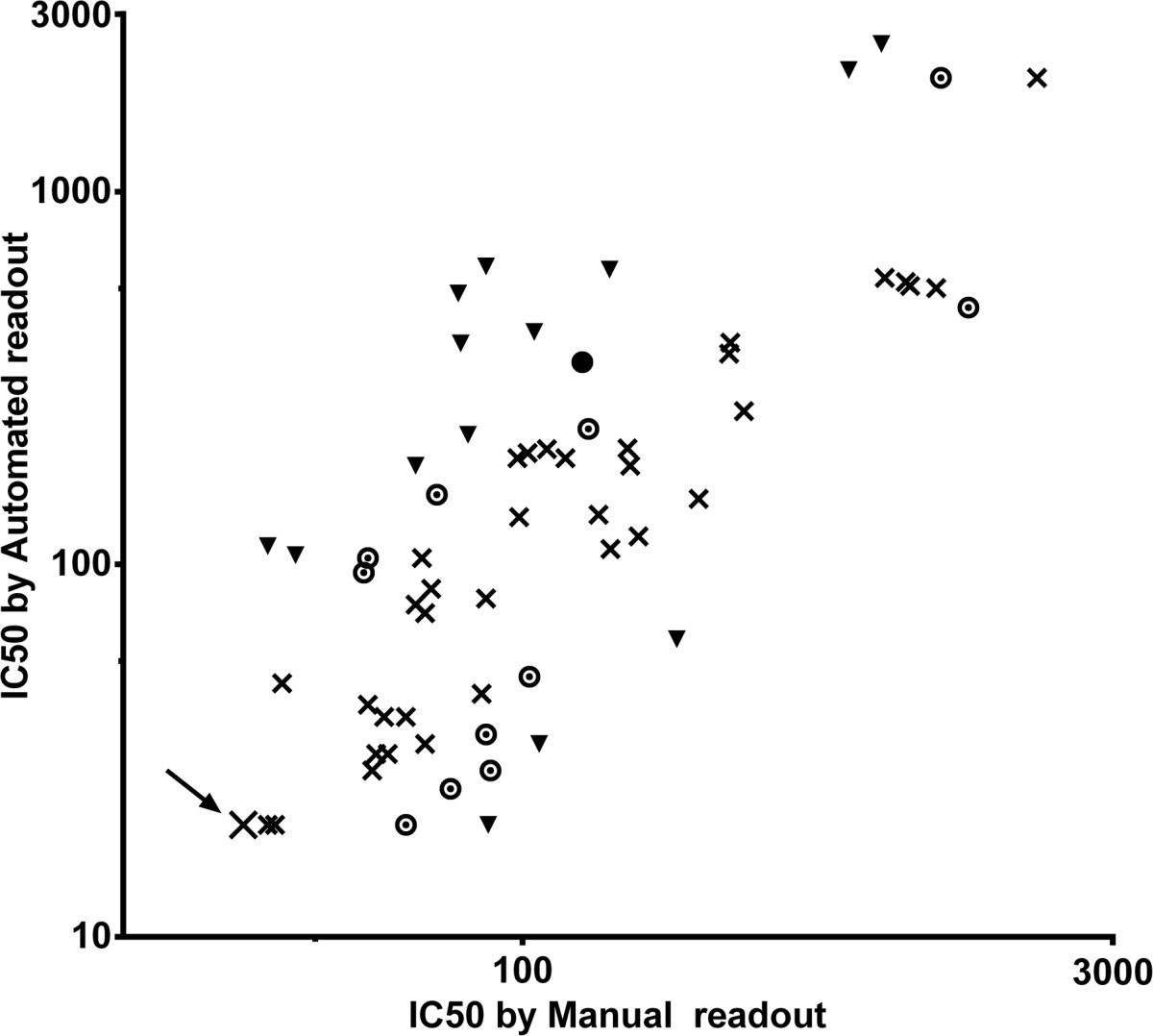
**Comparison of neutralization sensitivity (IC50) of manual and automated readout in 72 virus-plasma combinations, including eight with HIV-negative plasma (denoted with a big X and arrow).** The manual and automated readouts were from separate experiments. Readouts that do not vary more than 2-fold are denoted with x. Differences within a 2-3-fold range are depicted with a ring, and remaining virus-plasma combinations where the variation was >3-fold between manual and automatic readouts is depicted with a triangle. Triangle points are generally above the diagonal line indicating higher sensitivity of the automated readout. Statistically significant correlation between automated and manual readout according to Spearman rank (p < 0.0001, r = 0.8104).

We compared the manual and automated readouts using 72 virus-plasma combinations, including eight combinations with HIV-negative plasma (Figure 5). In 46 cases (64%), the manual and automatic readouts of 50% inhibitory concentration (IC50) were within twofold of each other and in 12 cases (17%) the difference was within a 2- to 3-fold range. This is an insignificant degree of variation in accordance with the accepted convention used in clinical virology (e.g., if serial 2-fold dilutions of antibodies are used). In the remaining 14 virus-plasma combinations (19%), variation of IC50 was more than 3-fold between manual and automatic readouts. Further examination of this latter group revealed that in 11 out of 14 combinations, the automatic readout was more sensitive than the manual readout in detecting neutralization (denoted as triangles above the diagonal line in Figure 5). These 14 points were not the result of an image analysis artifact, since no false positive background was detected with the negative plasma and the manual and automatic readout of other inhibitory reagent-virus combinations run on the same plate and day were in close agreement. Taken together, the APR assay detected neutralization similarly, but with generally increased sensitivity, as compared to the manual PR readout.

### Comparison of APR assay with other HIV-1 neutralization assays

**Figure 6:**
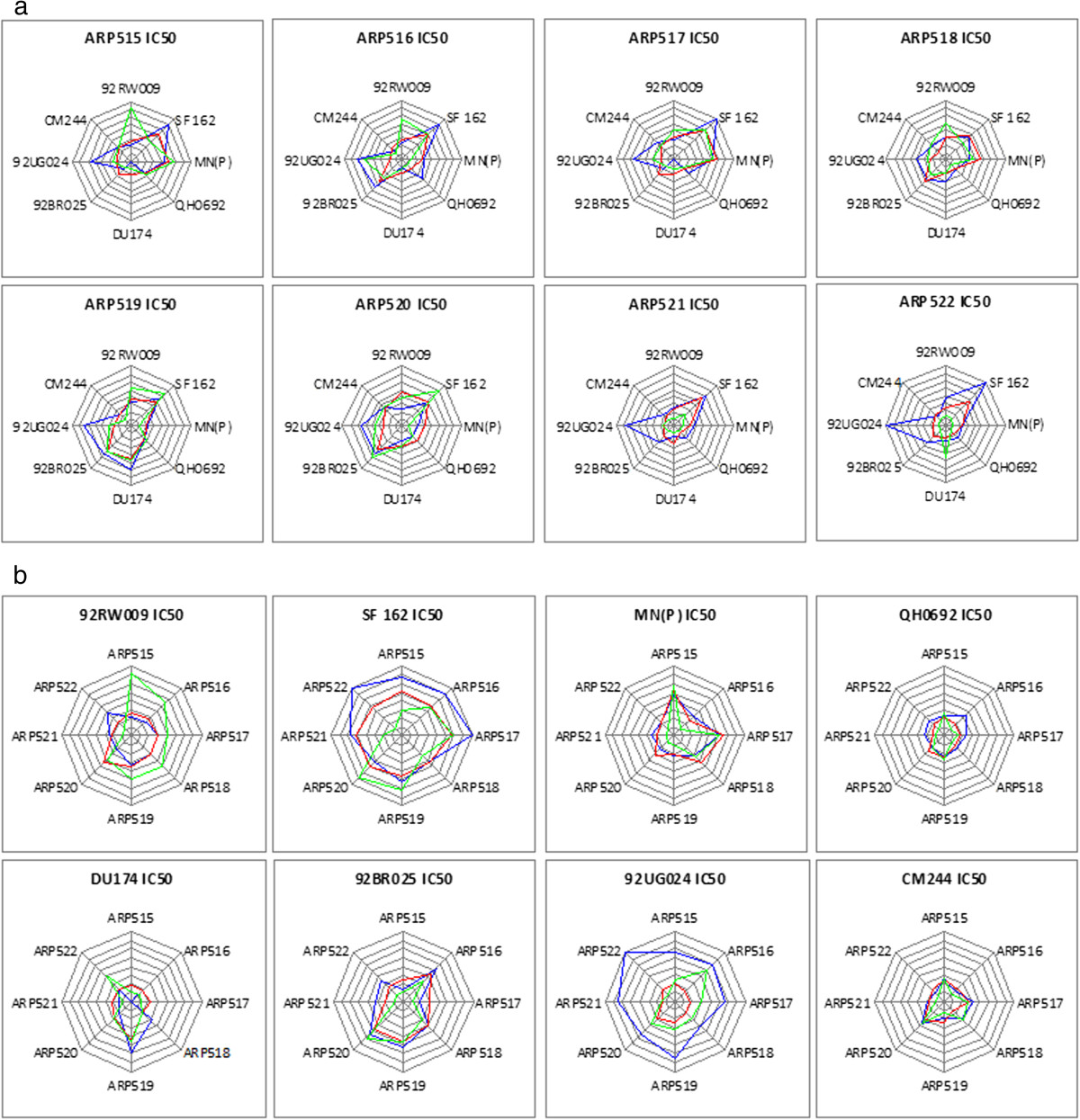
**Comparisons of neutralization results generated by the automated plaque reduction (APR) assay with that of pseudovirus (PSV) based and peripheral blood mononuclear cell (PBMC) based assays.** Circular “radar” plots of 50% inhibitory concentration-(IC50) values across **(a)** viruses and **(b)** plasma for the PSV assay (blue lines), the PBMC assay (red lines) and the APR assay (green lines). The radial axes extending from the plot center represent a particular **(a)** plasma and **(b)** virus for each axis. The radial scale is normalized, with the center representing no neutralization, the concentric grid lines representing 2-fold dilution steps and outermost lines representing the highest neutralization (IC50 > 1280). For each virus-plasma combination, the geometric mean IC value is calculated and plotted for each assay, with the points connected within a given assay.

The APR assay has been developed within the framework of an international network for comparison of HIV neutralization assays [3]. Comparisons on the IC50 level with other virus infection assays, including the PBMC and pseudovirus (PSV) assays using plasma and monoclonal antibodies, showed that sensitivities were dependent on both virus and plasma (Figure 6a and b). No statistical differences were found when comparing results of the APR and the PBMC-based assays when considering either virus neutralization sensitivity or plasma neutralization capacity. When comparing the APR and the PSV assays, virus neutralization sensitivity was statistically similar for seven out of eight viruses (Figure 6a); neutralization of 92UG024 was detected with higher sensitivity with the PSV assay as compared to both the APR assay (p < 0.05) and the PBMC assay (p < 0.001) (Figure 6a). For the neutralization capacity of plasma, results generated from the APR and the PSV assay were not significantly different with six out of eight plasma tested (Figure 6b). The exceptions were plasma ARP521 and ARP522, which displayed higher neutralization titers in the PSV assay compared with the APR assay (p < 0.01). We noted that it was particularly the use of ARP521 and ARP522 in combination with the 92UG024 and SF162 viruses that influenced the results. Overall, the sensitivity of the automatic PR assay was in the range of the other assays.

### High-content readout by the APR assay allows analysis of cell-cell fusion inhibition

**Figure 7:**
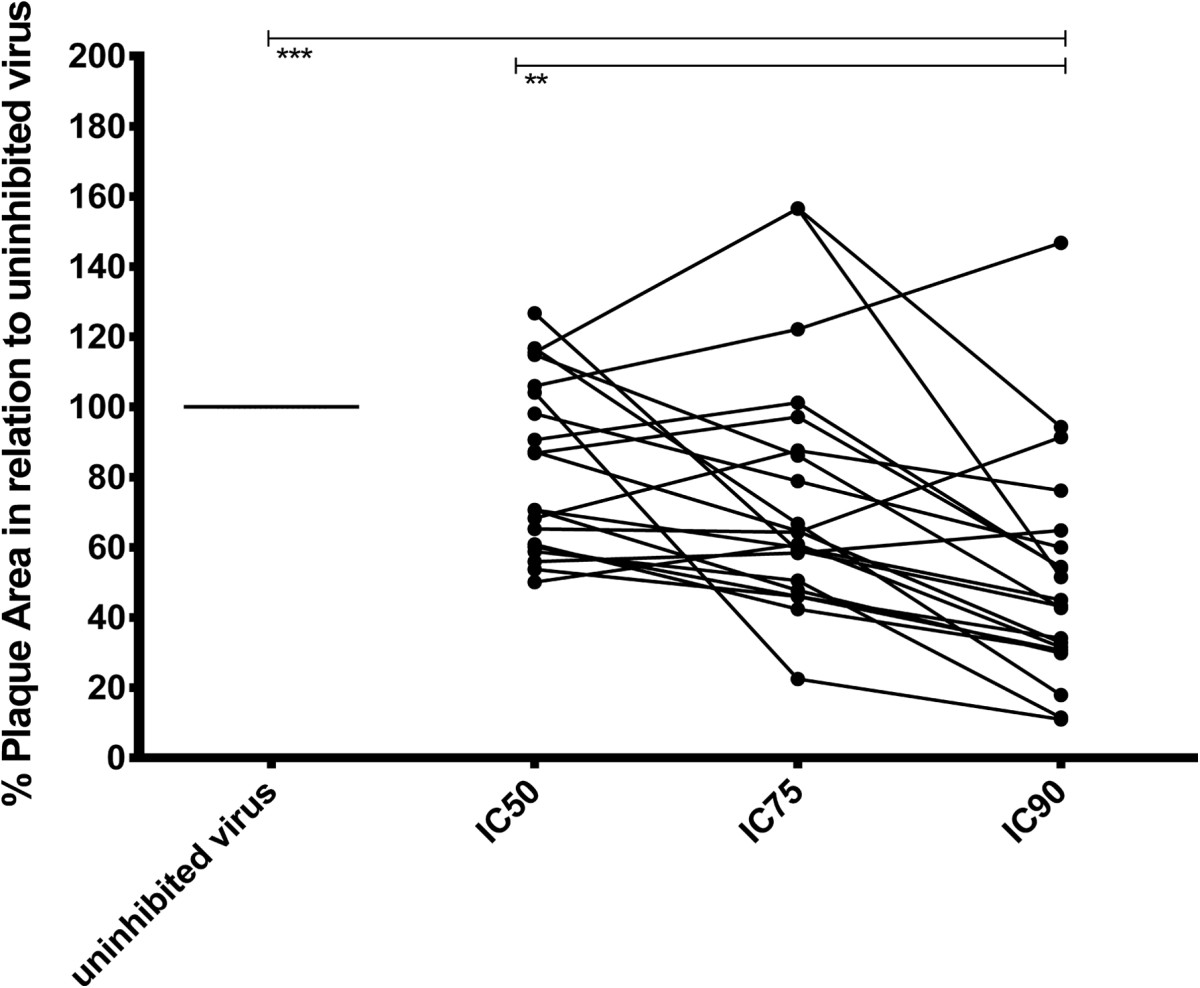
**Plaque area for different virus-plasma combinations and inhibitory reagent concentrations that reached IC90.** Percent plaque area relative to uninhibited virus (100) is shown for virus-plasma combinations where 50, 75 and 90% inhibitory concentrations (IC50, IC75 and IC90) could be determined. The plaque size of the same virus-plasma combinations at different IC values are connected. Statistically significant differences are indicated according to Friedman’s test and Dunn’s Post Test, **p < 0.01; ***p < 0.001.

**Figure 8:**
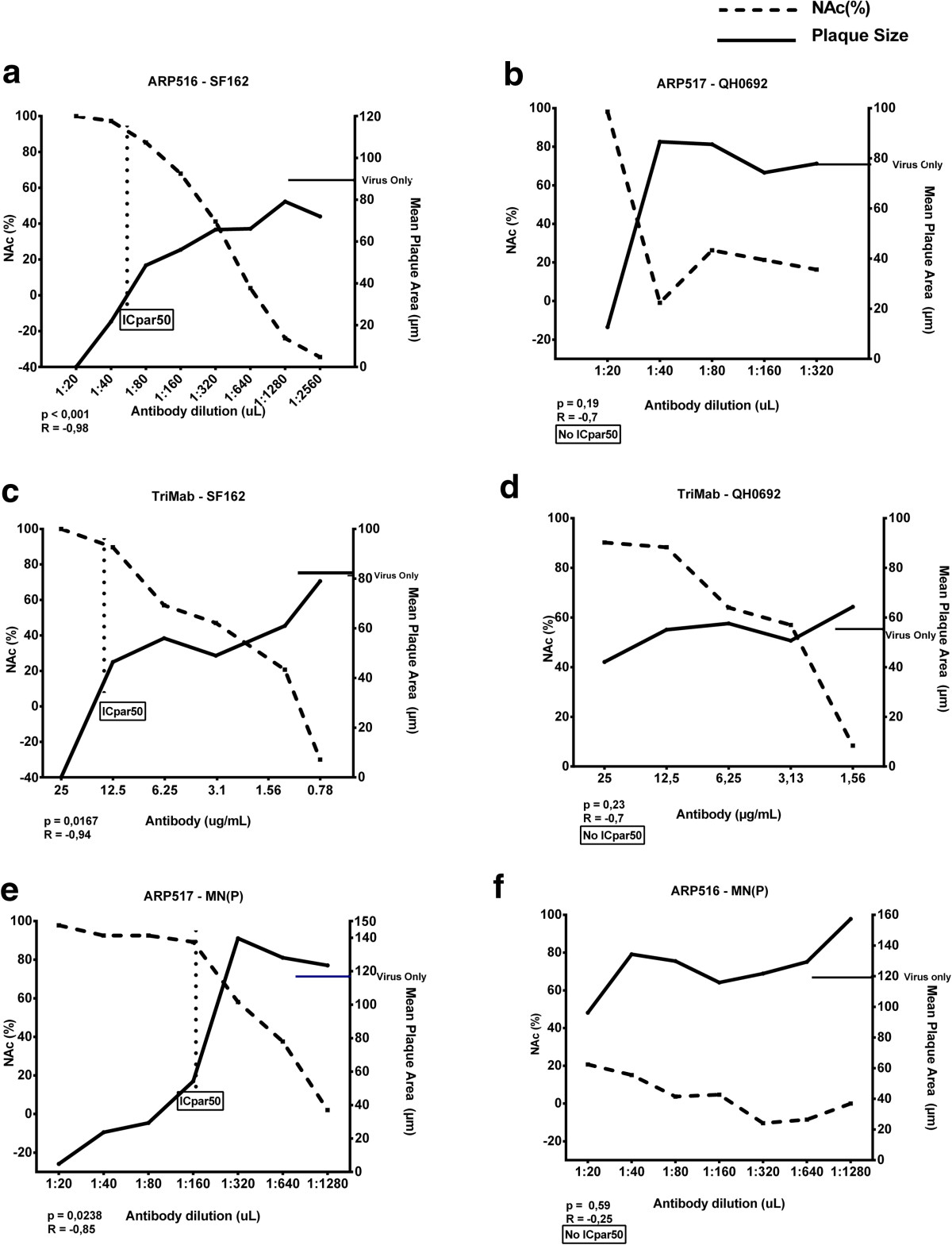
**Relationship between neutralization and plaque area.** ^_______^, mean plaque area (μm, y axis on the right) indicated with a solid line; -------, neutralization (NAc%, y axis on the left) indicated with a broken line in each panel and ICpar50 values are indicated with vertical dotted lines in panel **a**, **b** and **c**. The plaque area without addition of inhibitory reagent (virus only) is indicated by a horizontal line on the y axis on the right. Virus-plasma combinations: **(a)** ARP516-SF162; **(b)** ARP517-QH0692; **(c)** TriMab-SF162; **(d)** TriMab-QH0692; **(e)** ARP517-MN(P); **(f)** ARP516-MN(P). R and p values are given for the correlation of neutralization and plaque area according to Spearman rank.

We next evaluated the capacity of the APR assay to quantify the size of the plaque area, as a measure of cell-cell fusion. Changes in mean plaque area were evaluated in single virus-inhibitory reagent combinations. Results showed that quantification of plaque area was possible. Analysis of the relationship between neutralization level and plaque area across virus-inhibitory reagent combinations where IC50, IC75 and IC90 was achieved, revealed that the mean plaque area was significantly reduced at 90% inhibitory concentration (IC90) but not at IC50 (Figure 7), indicating that the level of neutralization seems to be important for inhibition of virus-mediated cell-cell fusion. Furthermore, the decrease in plaque area was statistically significant only when neutralization reached IC90 with plasma dilution exceeding 1:50 or an antibody concentration above 6 μg/mL (for TriMab). This was the case for four inhibitory reagent-virus combinations (Figure 8a, c, e and Additional file 1: Table S1).Fourteen combinations that reached IC90 with a plasma dilution lower than 1:50 and 11 combinations that reached IC50 showed a trend towards decreased plaque area but could not be statistically confirmed (exemplified in Figure 8b). Apparently, the steep fall in neutralizing activity upon dilution provided too few points for curve fitting. Twelve other inhibitory reagent-virus combinations that showed neutralization did not display reduction in mean plaque area (exemplified in Figure 8d). As expected, those inhibitory reagents lacking neutralizing activity against a particular virus did not affect the plaque area (e.g., Figure 8f). We did not identify a particular reagent capable of reducing plaque area of all viruses.

For quantitative analysis of plaque reduction we introduced a new nomenclature, ICpar (inhibitory concentration for plaque area reduction). ICpar indicates the concentration at which the antibody causes a decrease in plaque area, e.g., ICpar50 is a 50% reduction in plaque area. IC90 and ICpar50 values were similar for most virus-antibody combinations (Figure 8 and Additional file 1: Table S1) indicating that inhibition of cell-to-cell fusion requires higher quantities of inhibitory reagent than inhibition of virus cell entry and replication.

### Virus-induced cell-cell fusion characteristics based on plaque area readout

**Figure 9:**
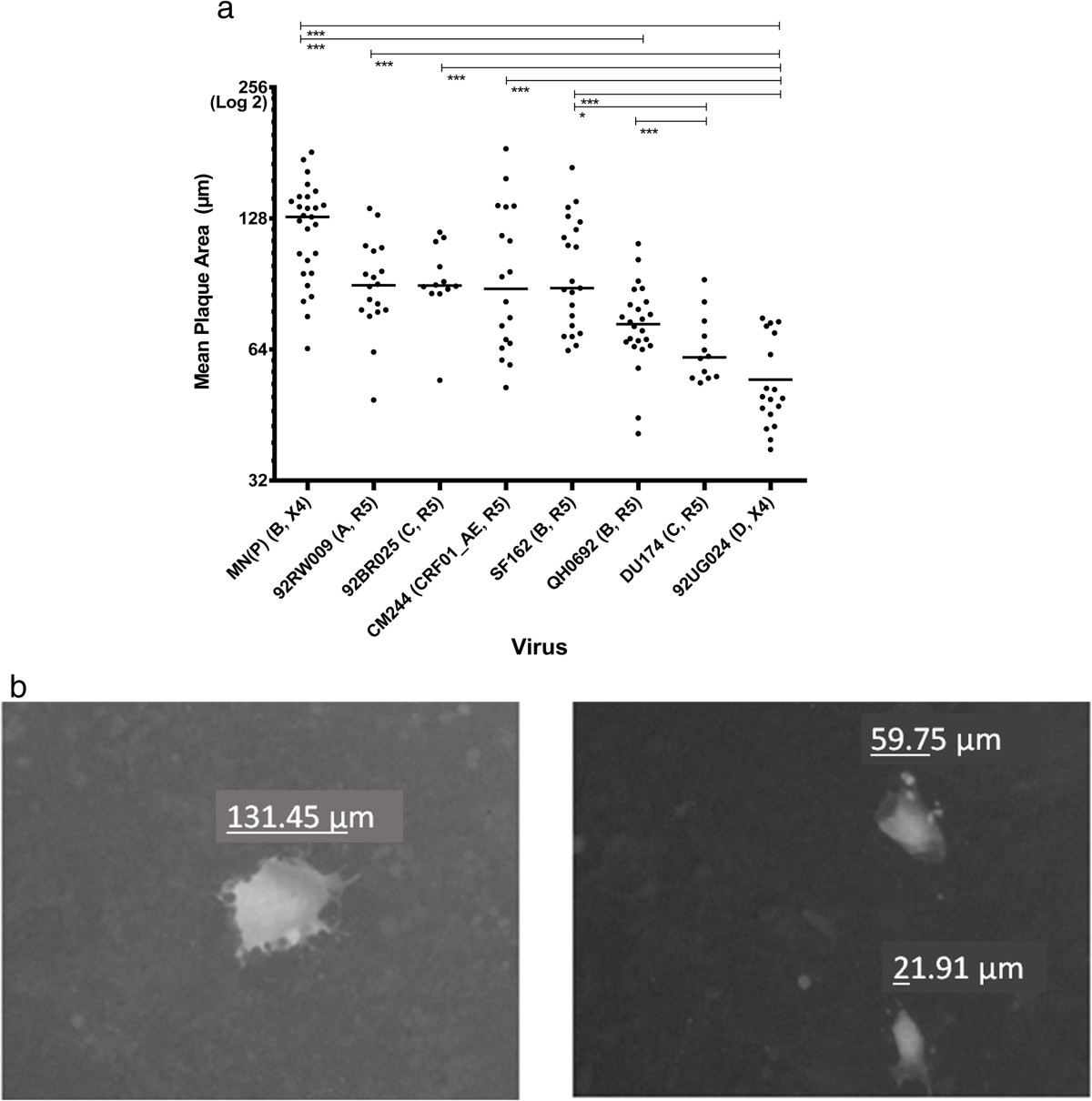
**HIV-1 isolates display different characteristic plaque size. (a)** Mean plaque area of uninhibited HIV-1 isolates with subtype and coreceptor use, indicated in parentheses. Each dot represents the mean plaque area per/well given in microns (Y axis). Horizontal bars indicate the mean of four to nine experiments run in triplicate wells. Statistically significant differences according to Kruskal-Wallis test and Dunn’s Post test. * < 0.05; *** < 0.001. **(b)** MN(P) exhibit larger plaques (left) while 92UG024 exhibit smaller plaques (right) regardless of virus titer.

We next analysed the size of the plaque area in relation to the different virus isolates. The mean plaque area of each virus yielded a characteristic size distribution (Figure 9), regardless of virus titration (data not shown). However, neither coreceptor use nor subtype of HIV-1 correlated to plaque area, as both X4 and R5 viruses and subtype B and C viruses were found across the size distribution. It is interesting to note that the 92UG024 virus, yielding relatively small plaques, was used in five of the 11 cases where automatic readout was more sensitive, suggesting that the small plaques may be more difficult to score by eye.

## Discussion

Here we describe a novel image-based methodology for assaying HIV neutralization in an automated high-content manner. Our high-content assay allows, for the first time, evaluation of both HIV neutralization and inhibition of cell-cell fusion within the same assay. This is achieved by quantifying the reduction in number of plaques and mean plaque area, respectively. The image analysis pipeline for the APR assay in GHOST(3) cells is based on the freely available CellProfiler software [12] and has now been optimized and adapted for automated reading. Automation offers many advantages: it is higher throughput, standardized, and inexpensive compared both to the manual PR assay and the classic virus infection assay performed in peripheral blood mononuclear cells (PBMC). Automated image analysis increases the objectivity of the plaque counting demonstrated by the very low intra-well variation among triplicate wells (data not shown) as well as low background in the negative controls.

A striking feature of the image-based approach is that it allows extraction of additional phenotypic features of viruses, including cell-cell fusion capacity. We observed differences between the eight HIV-1 isolates used in the present study. In fact, based on statistically significant differences in mean plaque area, viruses could be divided into two groups yielding large or small plaques. Large plaques may result from fusion of a higher number of cells than small plaques. It has been suggested that virus ability to mediate cell-cell fusion, i.e. syncytia, depends on the strength of the interaction of viral envelope protein with CD4 and coreceptors [14, 15]. In addition, Env clustering and mobility in the cell membrane may influence the outcome of syncytia formation [16].

Our results show that a higher concentration of inhibitory reagent was needed to get a reduction in plaque area than that needed for neutralization. This was previously proposed by Yee et al. [17] and given the explanation that the interaction of the envelope glycoproteins gp120/gp41 (Env) with cell membrane CD4 may be different during cell-cell fusion than during virus-cell membrane fusion [18]. It is plausible that the molecular organization and surface density of gp120/gp41 may be different in Env-expressing cells and HIV-1 virions. Indeed, Purtscher et al. [19] proposed, similarly to what we show, that elevated concentrations of antibody are necessary to inhibit fusion between infected and target cells, and suggested this to be due to higher levels of gp120/gp41 expressed on the surface of infected cells as compared to the viral envelope. Interestingly, it was recently shown, in an HIV-1-infected humanized mouse model, that infected T cells form contacts with uninfected cells, and when the frequency of these contacts was reduced, plasma viremia was significantly decreased, strongly suggesting a role for cell-cell contacts in systemic viral spread [20]*.* Thus, it is conceivable that cell-cell fusion inhibition capacity of an antibody has a role in mitigating pathogenesis and may also be required for an effective HIV vaccine.

Antibody assessment plays a central role in HIV-1 vaccine development where, analogous to other virus infections, virus neutralization is considered particularly important. Accordingly, international comparisons of a wide range of HIV-1 neutralization assays have been performed and showed that no assay alone detects neutralization over the entire spectrum of virus-reagent combinations [2, 21]. The APR assay has also been standardized and compared with other HIV neutralization assays within the framework of an international collaboration, Neutnet [2, 3]. As with the other assays, the sensitivity of our assay was dependent on both the neutralizing reagent and the virus. Thus the APR assay can be considered an information-rich alternative to the PBMC and PSV assays.

## Conclusions

Here we report on a novel image-based automated plaque-reduction assay where both HIV neutralization and inhibition of cell-cell fusion can be analyzed, for the first time within the boundaries of the same assay format. This high-content assay may be used as a tool for evaluation of multiple antibody effector functions, by ways of quantifying the reduction in number of plaques and mean plaque area, respectively. The image analysis platform described herein can be further developed with the potential to study additional features of antibody-virus-cell interactions, which may prove important in antibody-based HIV vaccine design.

## Electronic supplementary material

Additional file 1: Table S1: High-throughput, automated, image-based assay for HIV neutralization by plaque reduction. (DOCX 597 KB)

Below are the links to the authors’ original submitted files for images.

Authors’ original file for figure 1

Authors’ original file for figure 2

Authors’ original file for figure 3

Authors’ original file for figure 4

Authors’ original file for figure 5

Authors’ original file for figure 6

Authors’ original file for figure 7

Authors’ original file for figure 8

Authors’ original file for figure 9

Authors’ original file for figure 10

Authors’ original file for figure 11
