# Supplementary material for: Automated image-based assay for evaluation of HIV neutralization and cell-to-cell fusion inhibition
Source: BMC Infect Dis. 2014 Aug 30;14:472. doi: 10.1186/1471-2334-14-472 (PMC4261578; doi:10.1186/1471-2334-14-472)
Supplement: Supplementary file 1 — Additional file 1: Table S1: High-throughput, automated, image-based assay for HIV neutralization by plaque reduction. (DOCX 597 KB) [file 12879_2014_4048_MOESM1_ESM.docx]

**Additional file 1**

**High-throughput, automated, image-based assay for HIV neutralization by plaque reduction**

Enas Sheik-Khalil1, Mark-Anthony Bray2, Gülsen Özkaya Şahin1, Gabriella Scarlatti3, Marianne

Jansson1, Anne E. Carpenter2 and Eva Maria Fenyö1

| Supplementary Table 1 | Virus-inhibitory reagent combinations that  reached IC90 in neutralization and antibody concentrations for 50% of plaque area reduction (ICpar50). |
| --- | --- |
| Supplementary Methods: | How to get started with the APR |
| Supplementary Note 1: | Imaging equipment and settings |

**Supplementary Table 1.** Virus-inhibitory reagent combinations that reached IC90 in neutralization and antibody concentrations for 50% plaque area reduction (ICpar50).


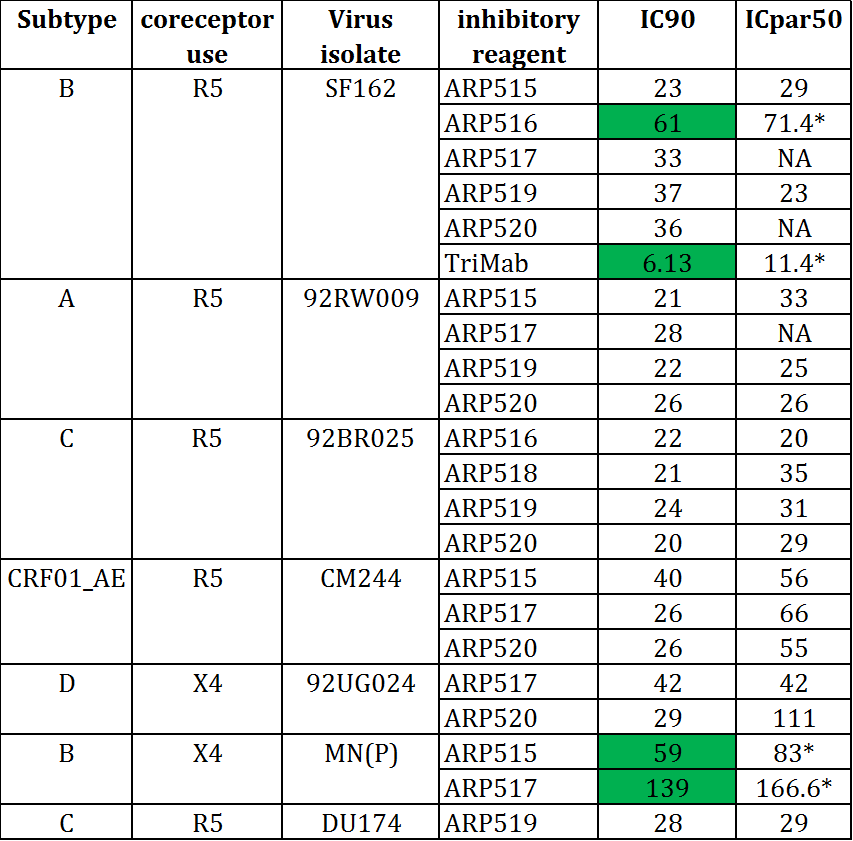


Neutralization reactions that reached IC90 with an antibody dilution exceeding 1:50 (above 6µg/mL for TriMab) are highlighted in green. Statistically significant correlation between neutralization and plaque area according to Mann Whitney <0.05 is denoted with *. IC90 and ICpar50 values were similar for most virus-antibody combinations, with the exception of the 92UG024-ARP520 combination.

**Supplementary Methods**

*How to get started with the automated plaque reduction (APR) assay*

The APR assay uses a set of CellProfiler modules specific for HIV neutralization determination by plaque reduction in GHOST(3) cells. A pipeline consists of a series of modules. In order to get started, you simply download and install the CellProfiler software, download the APR pipeline, and adjust it to analyze your own image data.

1. Download and install CellProfiler 2.1 from [http://www.cellprofiler.or](http://www.cellprofiler.org/)g
2. Download the APR pipeline from <http://cellprofiler.org/published_pipelines.shtml>for HIV neutralization analysis.
3. Double-click the file *APRassay_2_DetectQuantifyPlaques.cppipe* to start CellProfiler and load the pipeline.
4. The initial module selected will be the Images modules, in which you provide CellProfiler with the input images. Drag-and-drop the images (or folder containing your images) into the space indicated by “Drop files and folders here”.


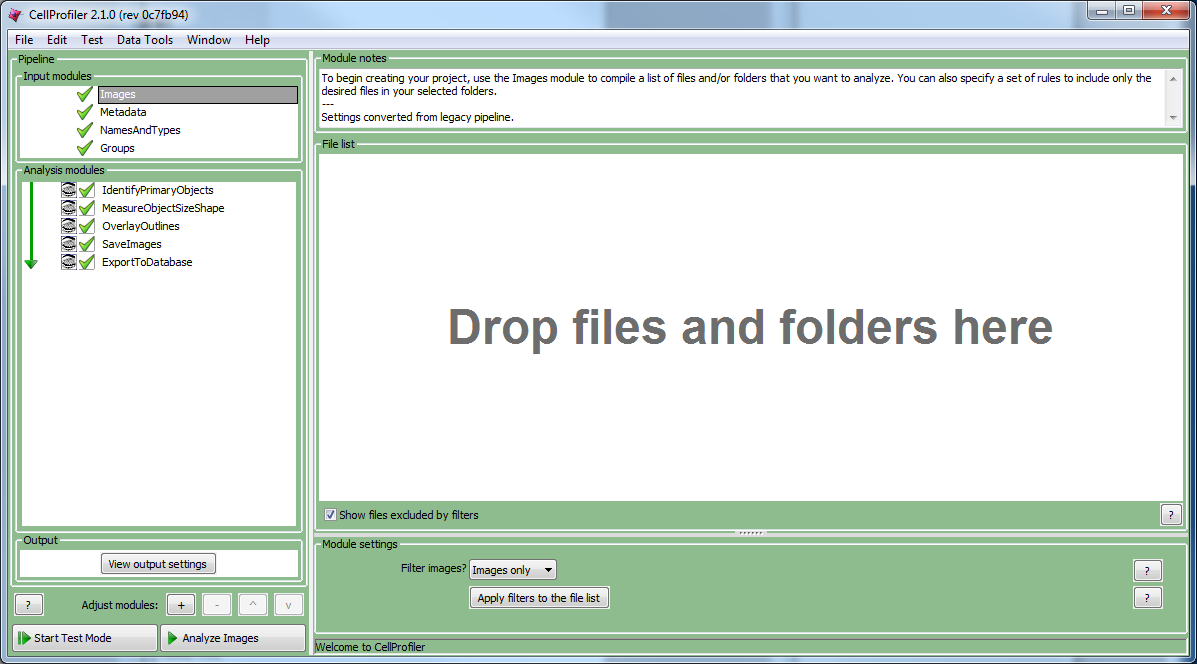


1. Select *Window > Show all windows on run* from the main menu; the “eye” icons next to the module name in the pipeline panel will change from closed to open (4).


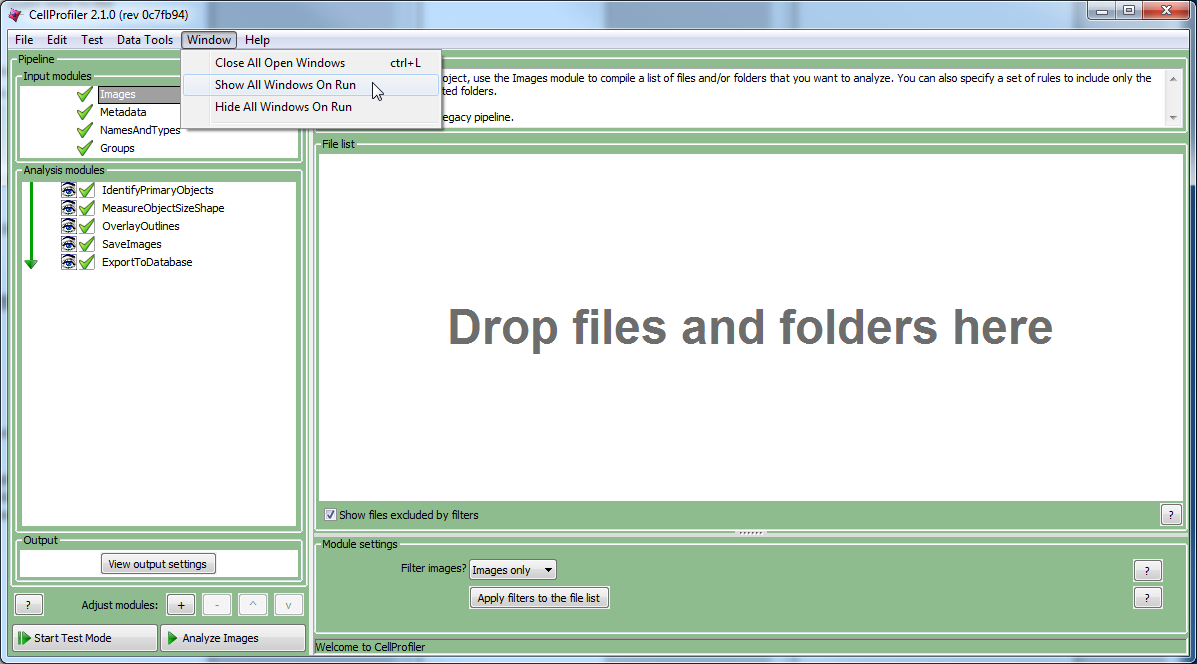

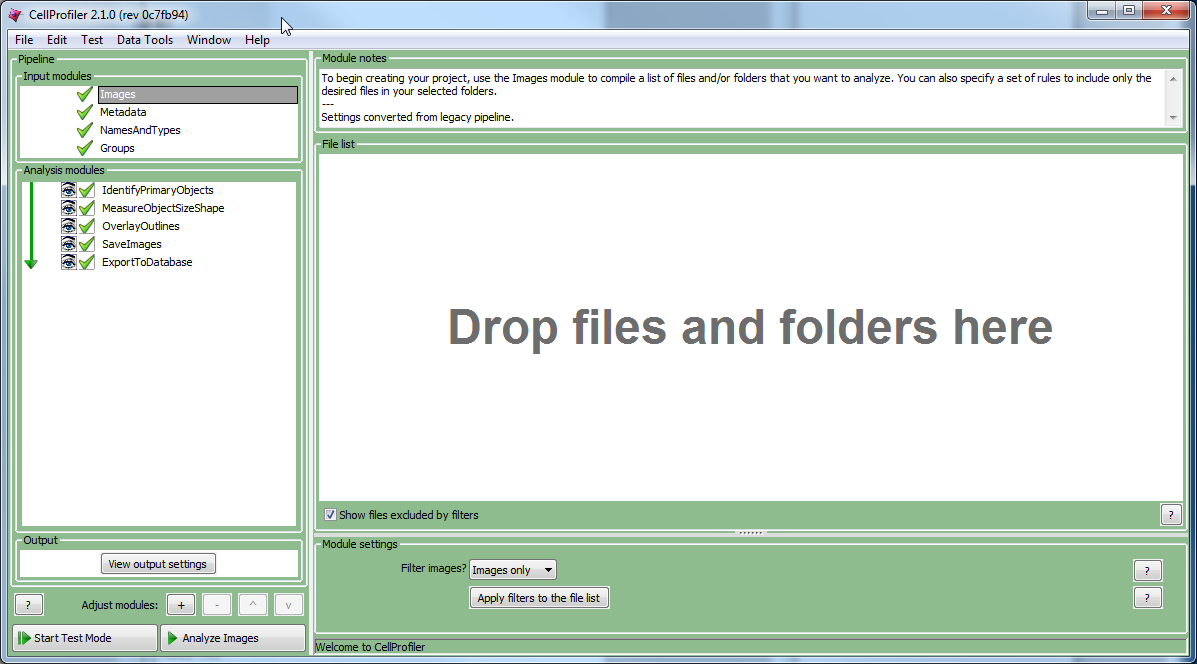


1. Click the “Start Test Mode” button at the bottom-left to enter CellProfiler’s test mode. This mode will allow you to step sequentially through each module by clicking the “Step” button. As each module is executed, the results are shown in a display window. We recommend using this mode to further tune the module settings to appropriate values for your assay. Exit Test mode by clicking the “Exit test mode” button in order to run the full image data set.


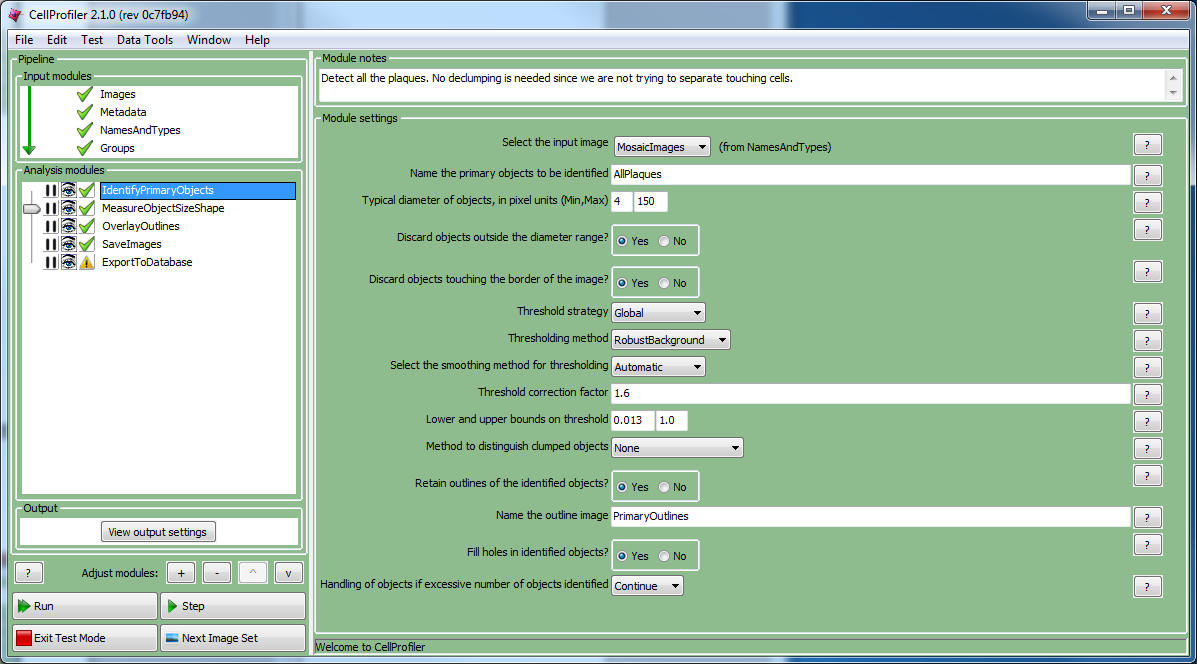


1. Click the “View output settings” button to set the Default Output Folder, which is the location of the output produced by the pipeline.


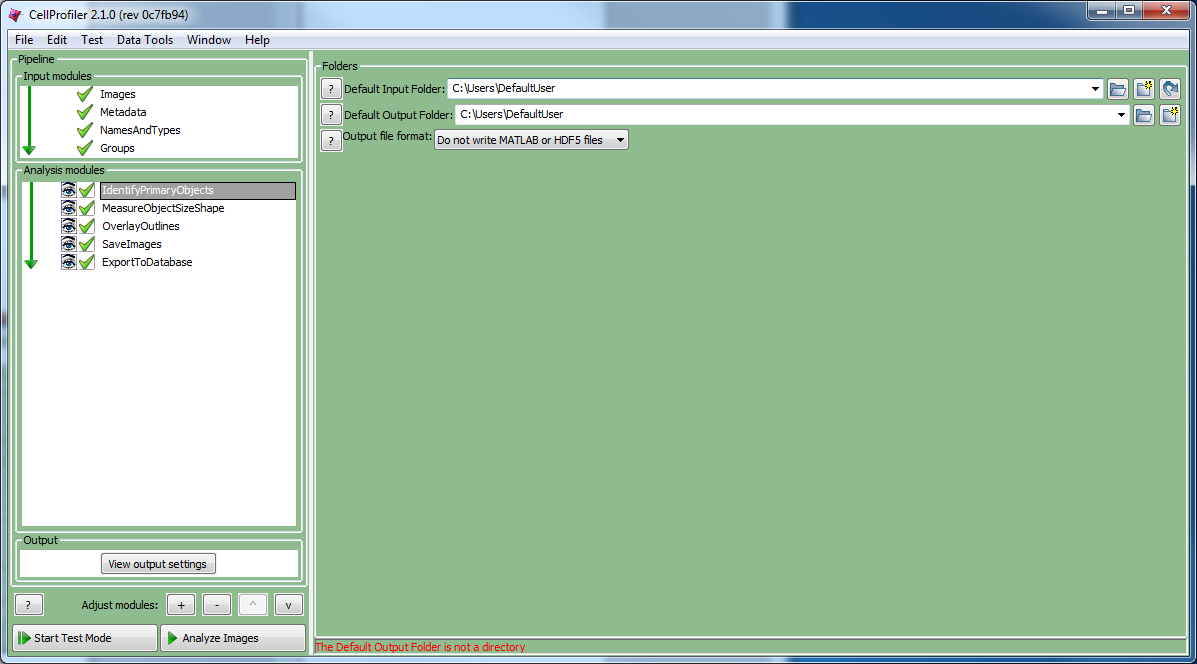


1. Click the “Analyze Images” button (located in the bottom left) to run the full pipeline on the images you provided via the Images module. A module display window will open for each module of the pipeline. Two comma-delimited (CSV) files containing the computed measurements will be written to your Default Output Folder.
2. The APR workflow consists of two pipelines. To run the pipelines sequentially rather than separately, select *File > Run Multiple Pipelines* from the menu bar.
3. Browse for the folder containing the APR pipelines and then click on the “Add” button to add the pipelines for sequential processing. Set the Default Input Folder and Default Output Folder to the folder where your images are located.


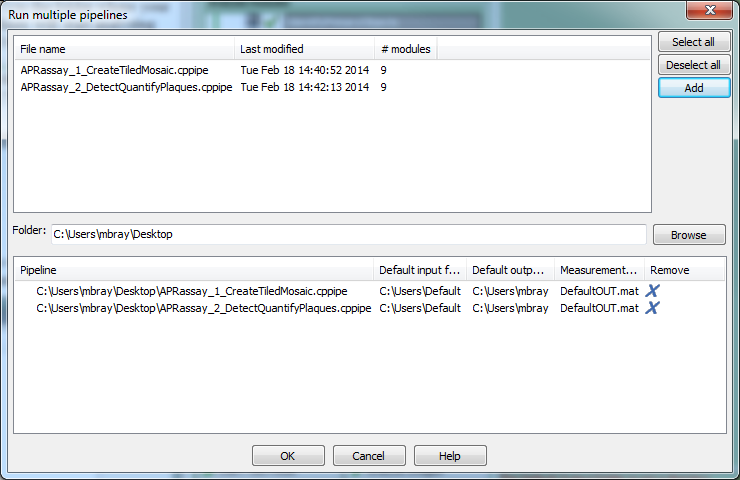


1. Upon clicking the “OK” button, the pipelines will start analyzing your images in sequential order.
2. In the first APR pipeline, if acquiring the images in meander mode, make sure to check the box for the "Tile in meander mode" setting in the *Tile* module.

For further information on how to adapt a pipeline to your own images, visit [http://www.cellprofiler.o](http://www.cellprofiler.org/)rg. Questions on using CellProfiler can be posted in the online moderated forum at http://www.cellprofiler.org/forum/.

**Supplementary Note 1: Imaging equipment and settings**

• Equipment: AxioObserver Z1 (Carl Zeiss AB, Sweden) equipped with an HBO light source (Carl Zeiss AB, Sweden). A Zeiss Neofluar Objective 10X/0.3 was used for all imaging with a large chip CCD camera (2/3") option.

• Image size: 1050x1300 pixels

• Pixel size: approx. 1µm

• Temperature: room temperature

• Cell culture: 96-well plates, Dulbecco’s Minimum Essential Medium (DMEM)

supplemented with 10% fetal calf serum (FCS) and antibiotics.

• Filters: two-color DAPI/GFP fluorescence filter set.

• Imaging modality: Nuclei were imaged in the DAPI channel for Hoechst-stained samples while GFP was imaged in the GFP channel

• Exposure time: 200 ms

• Processing of 96-well plates before imaging: remove media, treat cells with Hoechst 33342 for 20 minutes, wash with PBS then seal plates with aluminum foil.

• Further manipulation: none
