# Supplementary figures and images for: Automated image-based assay for evaluation of HIV neutralization and cell-to-cell fusion inhibition
Source: BMC Infect Dis. 2014 Aug 30;14:472. doi: 10.1186/1471-2334-14-472 (PMC4261578; doi:10.1186/1471-2334-14-472)

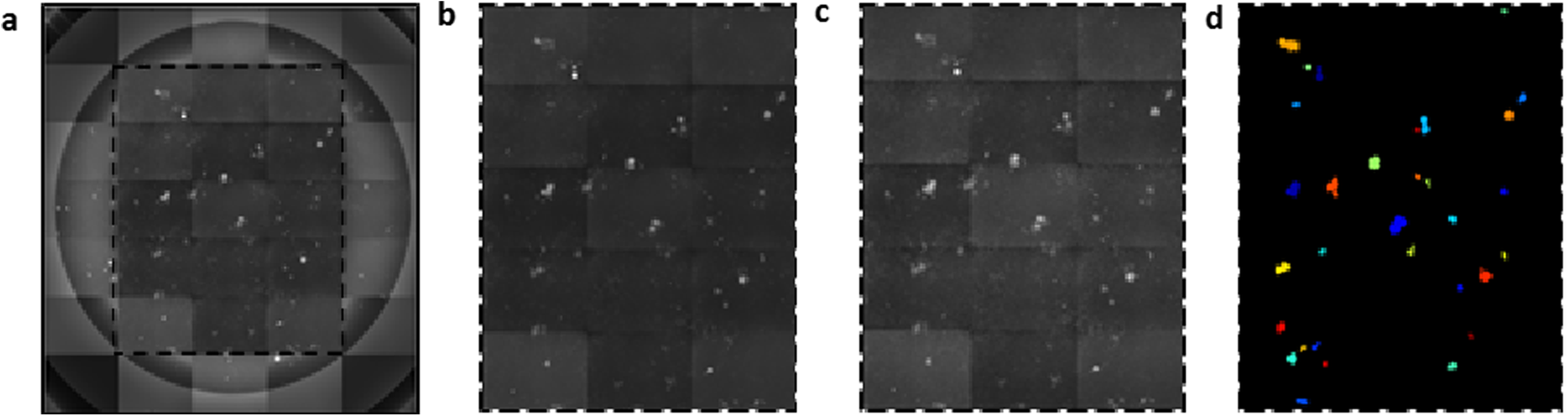

Supplement: Supplementary file 2 — Authors’ original file for figure 1 [file 12879_2014_4048_MOESM2_ESM.tiff]

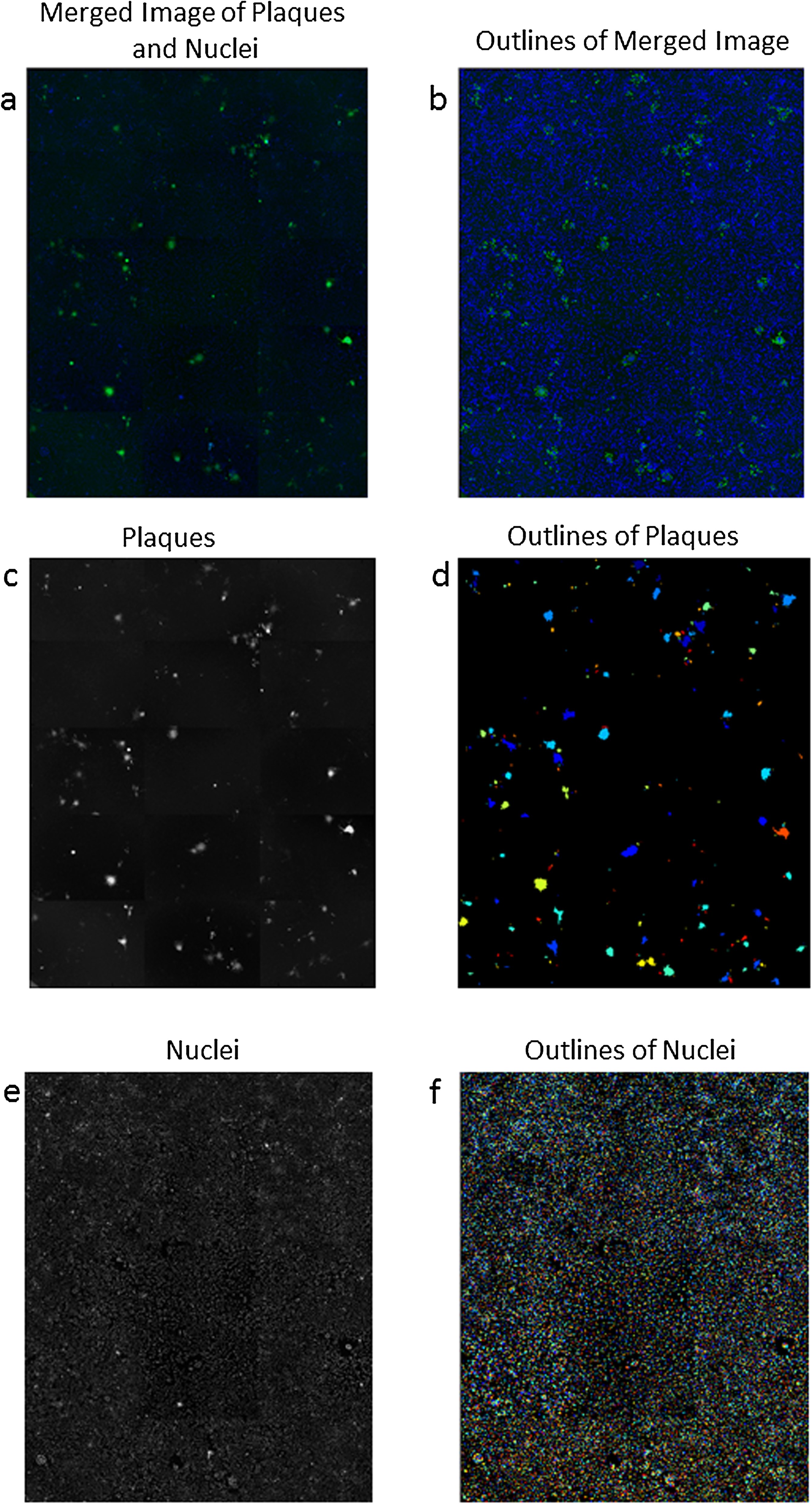

Supplement: Supplementary file 3 — Authors’ original file for figure 2 [file 12879_2014_4048_MOESM3_ESM.tiff]

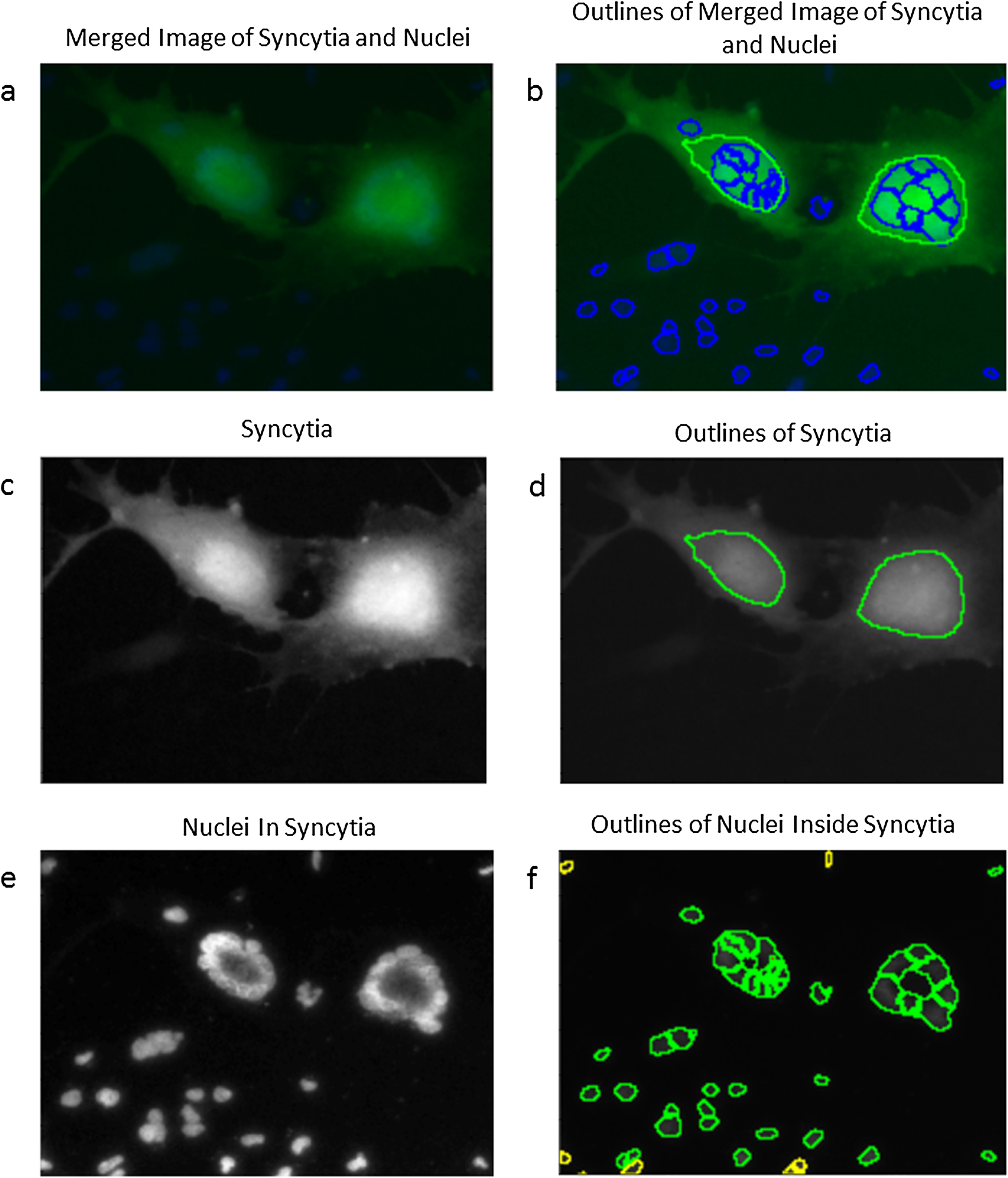

Supplement: Supplementary file 4 — Authors’ original file for figure 3 [file 12879_2014_4048_MOESM4_ESM.tiff]

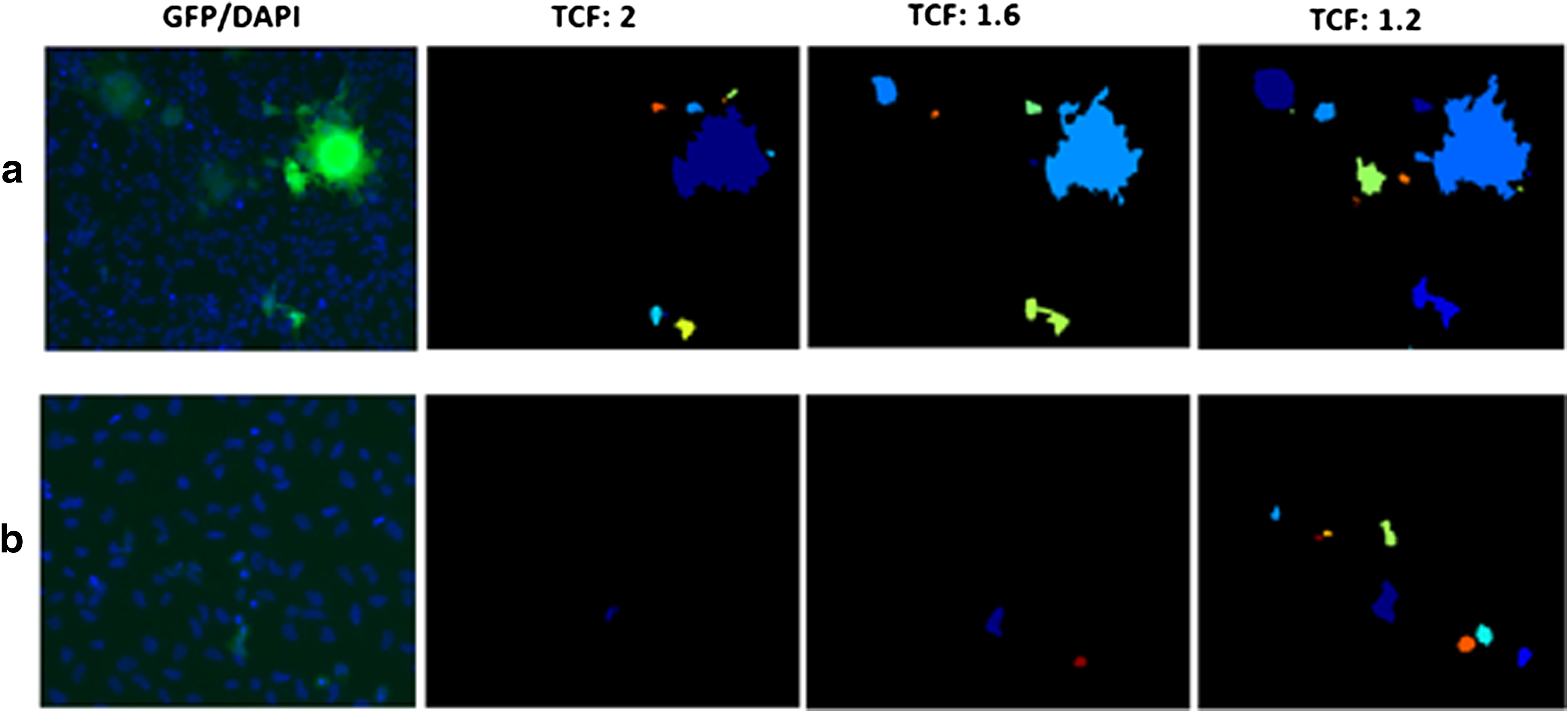

Supplement: Supplementary file 5 — Authors’ original file for figure 4 [file 12879_2014_4048_MOESM5_ESM.tiff]

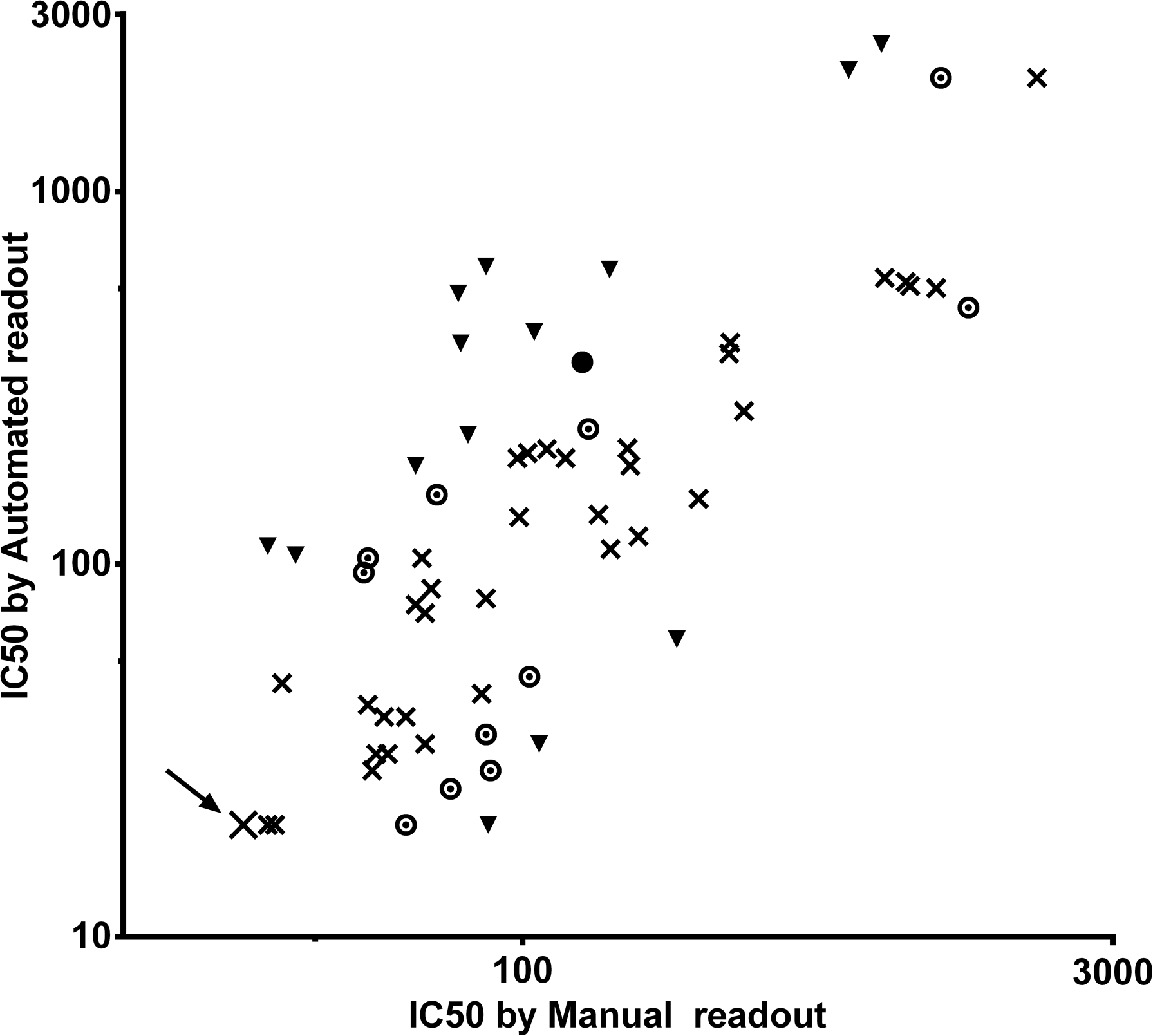

Supplement: Supplementary file 6 — Authors’ original file for figure 5 [file 12879_2014_4048_MOESM6_ESM.tiff]

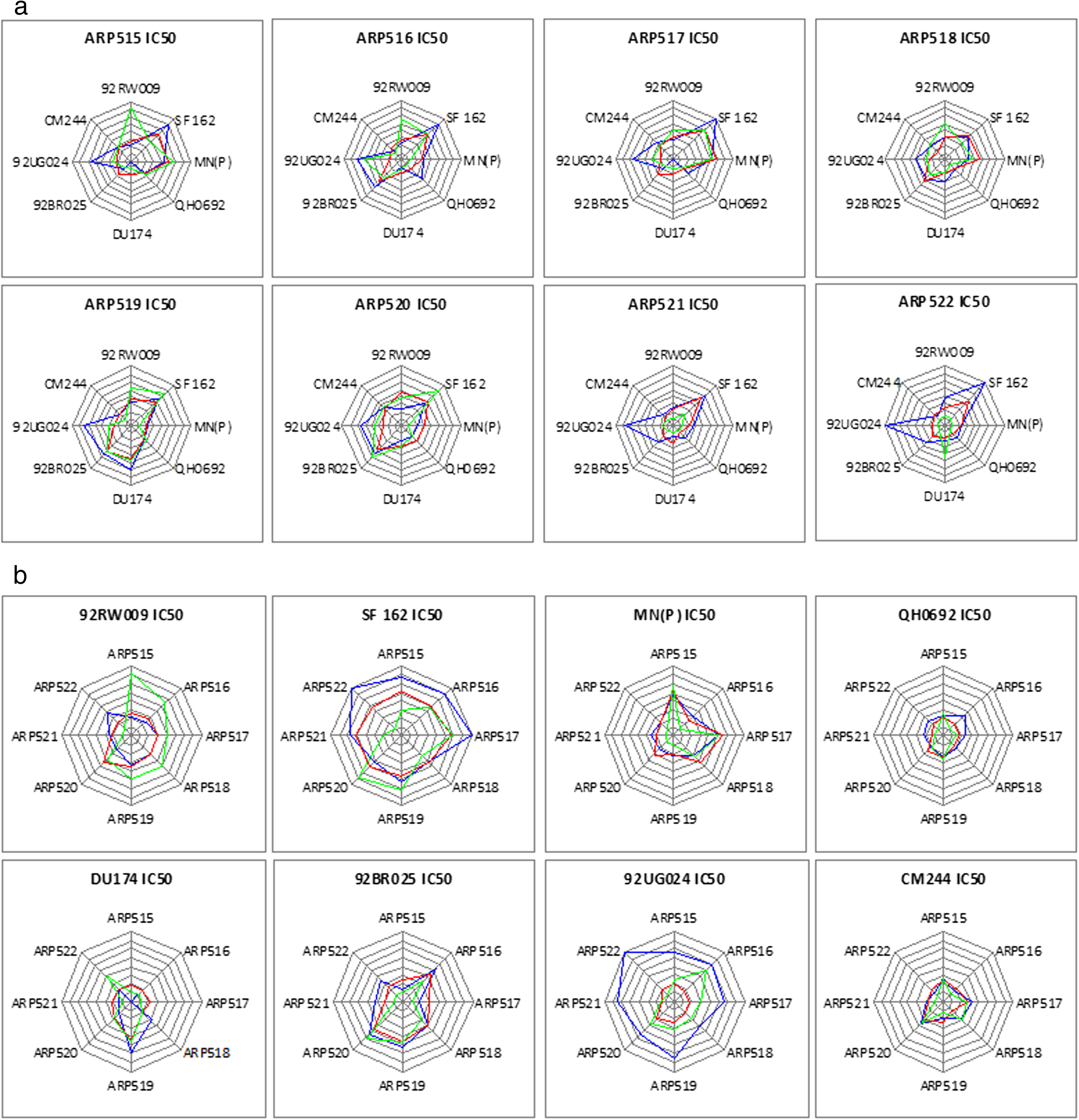

Supplement: Supplementary file 7 — Authors’ original file for figure 6 [file 12879_2014_4048_MOESM7_ESM.tiff]

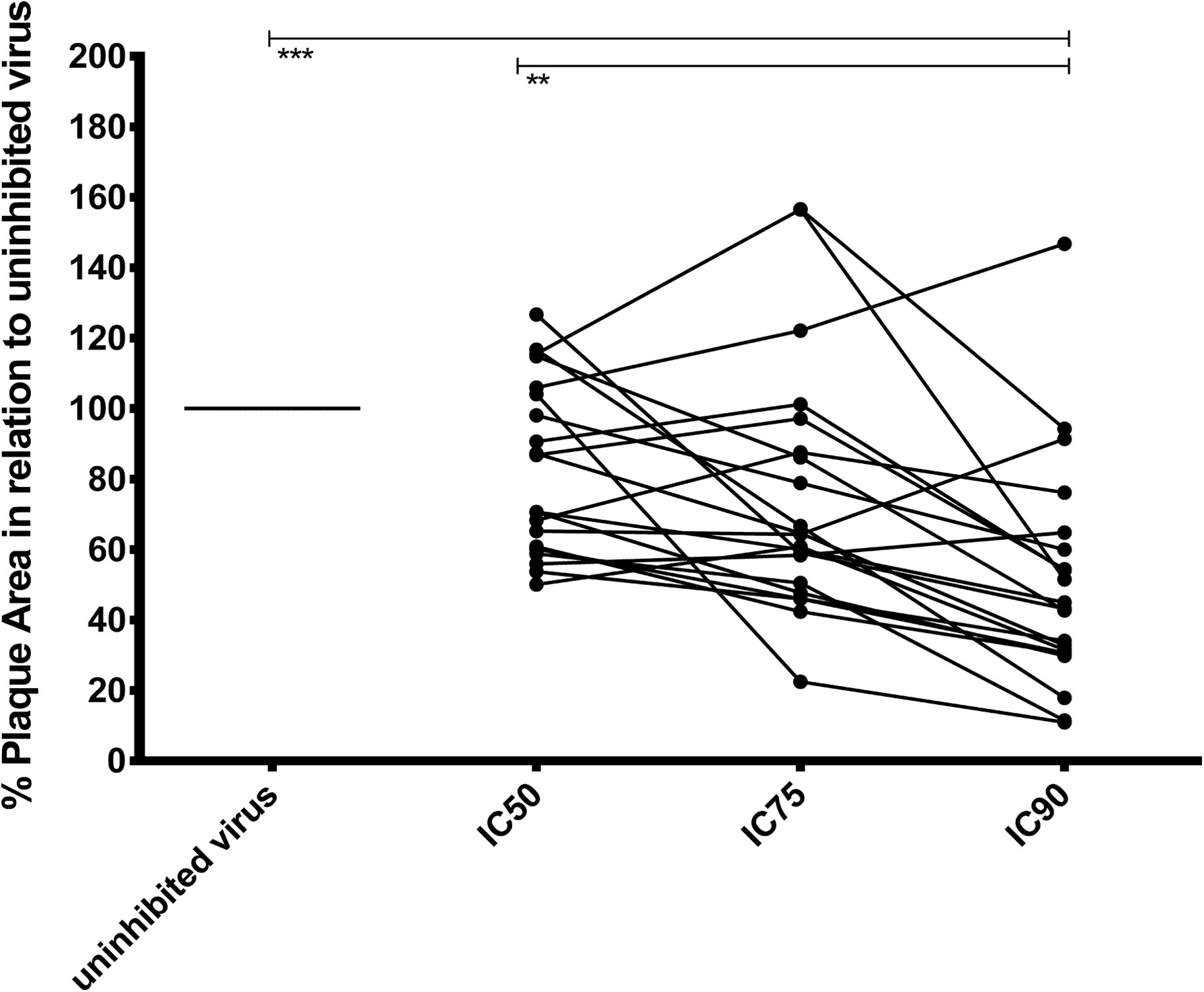

Supplement: Supplementary file 8 — Authors’ original file for figure 7 [file 12879_2014_4048_MOESM8_ESM.tiff]

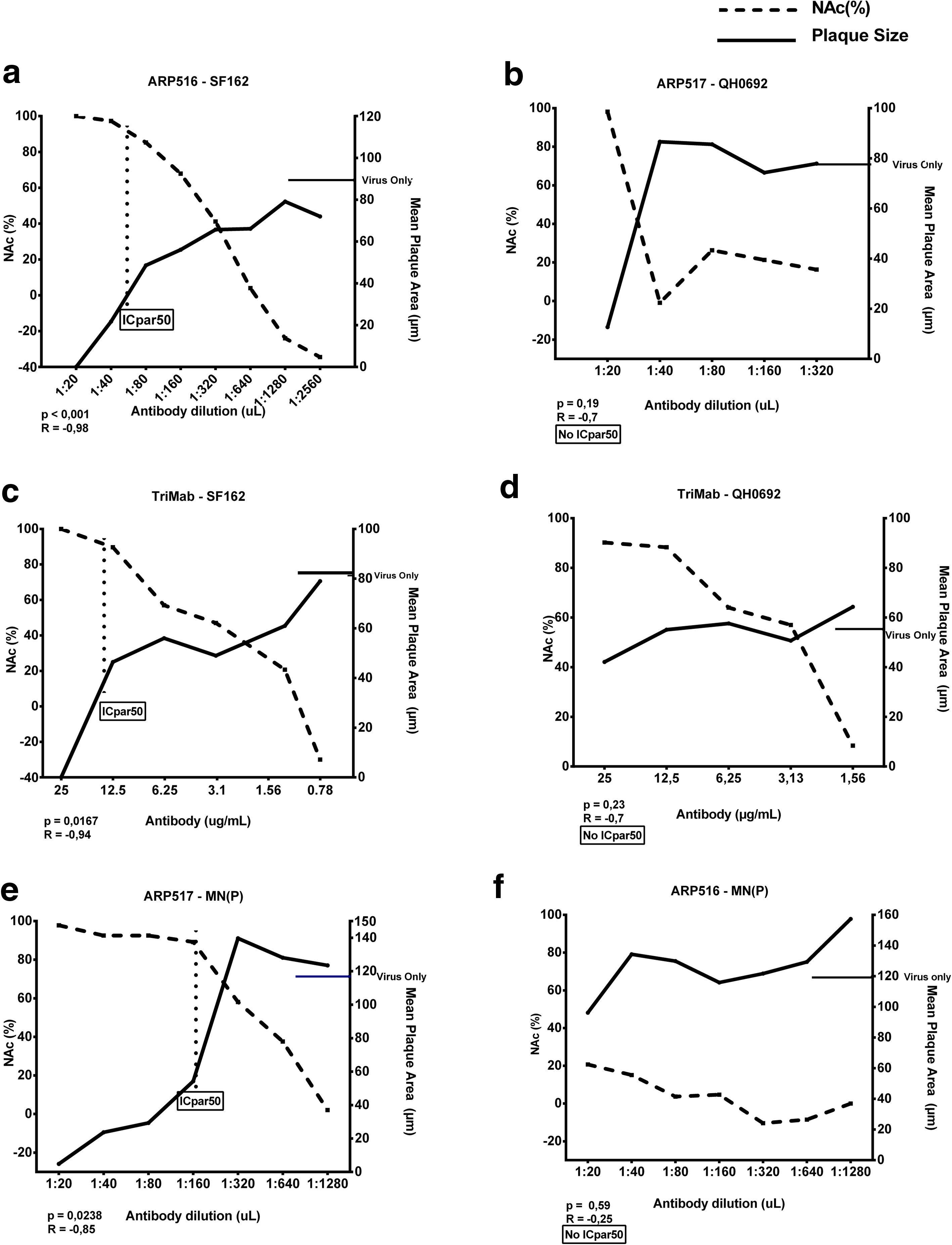

Supplement: Supplementary file 9 — Authors’ original file for figure 8 [file 12879_2014_4048_MOESM9_ESM.tiff]

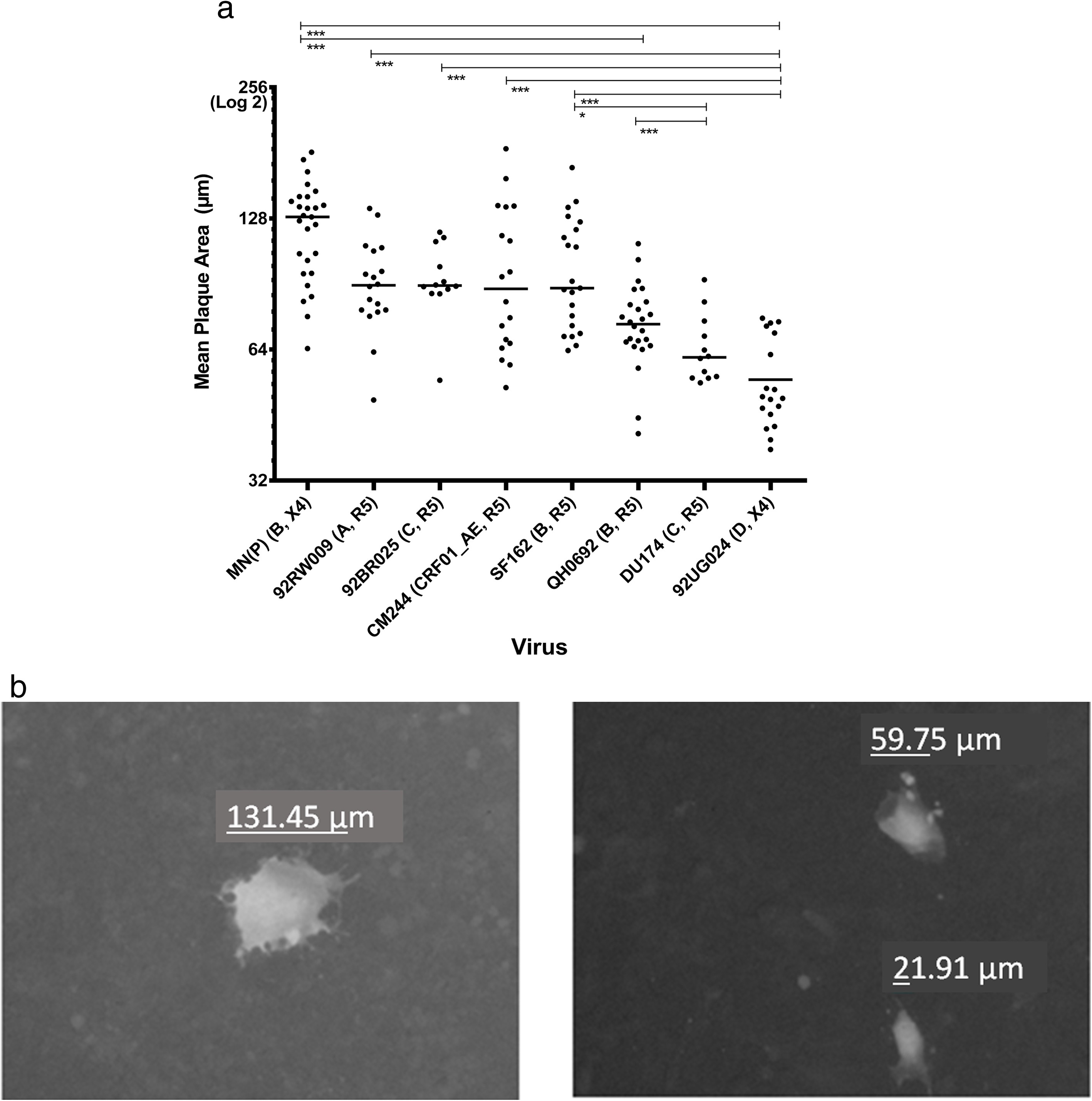

Supplement: Supplementary file 10 — Authors’ original file for figure 9 [file 12879_2014_4048_MOESM10_ESM.tiff]

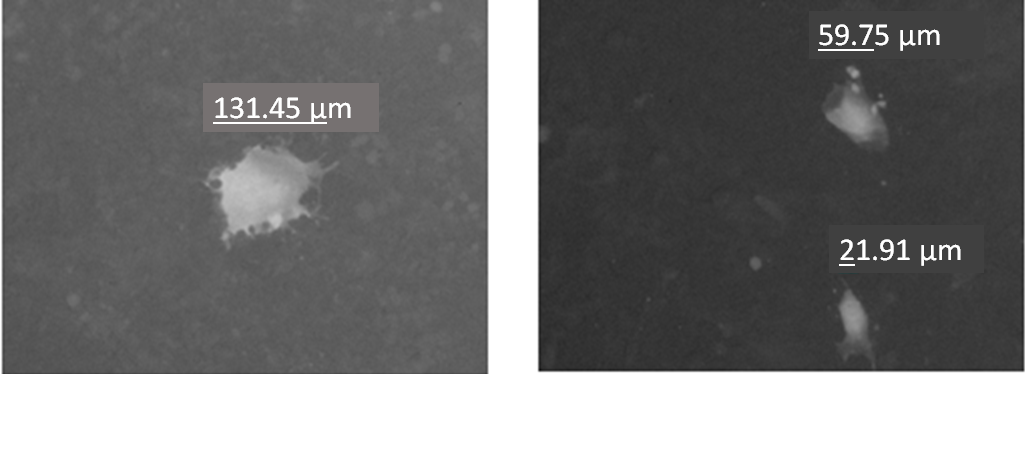

Supplement: Supplementary file 12 — Authors’ original file for figure 11 [file 12879_2014_4048_MOESM12_ESM.tiff]
